# CPMI-ChatGLM: parameter-efficient fine-tuning ChatGLM with Chinese patent medicine instructions

**DOI:** 10.1038/s41598-024-56874-w

**Published:** 2024-03-16

**Authors:** Can Liu, Kaijie Sun, Qingqing Zhou, Yuchen Duan, Jianhua Shu, Hongxing Kan, Zongyun Gu, Jili Hu

**Affiliations:** 1 School of Medical Informatics Engineering, Anhui University of Traditional Chinese Medicine, Hefei, 230012 China; 2 Anhui Computer Application Research Institute of Chinese Medicine, China Academy of Chinese Medical Sciences, Hefei, 230012 China

**Keywords:** Computer science, Biomedical engineering

## Abstract

Chinese patent medicine (CPM) is a typical type of traditional Chinese medicine (TCM) preparation that uses Chinese herbs as raw materials and is an important means of treating diseases in TCM. Chinese patent medicine instructions (CPMI) serve as a guide for patients to use drugs safely and effectively. In this study, we apply a pre-trained language model to the domain of CPM. We have meticulously assembled, processed, and released the first CPMI dataset and fine-tuned the ChatGLM-6B base model, resulting in the development of CPMI-ChatGLM. We employed consumer-grade graphics cards for parameter-efficient fine-tuning and investigated the impact of LoRA and P-Tuning v2, as well as different data scales and instruction data settings on model performance. We evaluated CPMI-ChatGLM using BLEU, ROUGE, and BARTScore metrics. Our model achieved scores of 0.7641, 0.8188, 0.7738, 0.8107, and − 2.4786 on the BLEU-4, ROUGE-1, ROUGE-2, ROUGE-L and BARTScore metrics, respectively. In comparison experiments and human evaluation with four large language models of similar parameter scales, CPMI-ChatGLM demonstrated state-of-the-art performance. CPMI-ChatGLM demonstrates commendable proficiency in CPM recommendations, making it a promising tool for auxiliary diagnosis and treatment. Furthermore, the various attributes in the CPMI dataset can be used for data mining and analysis, providing practical application value and research significance.

## Introduction

Traditional Chinese medicine (TCM) is an integral part of the Chinese cultural heritage, with a history of five thousand years and rich clinical experience, and is an important means of treating many diseases^1,2^. With the development of modern scientific technology, an increasing number of studies have confirmed the pharmacological effects and therapeutic efficacy of Chinese herbs, making TCM a research hotspot worldwide^3–5^. The World Health Organization (WHO) emphasized TCM as a popular and effective complementary and alternative medicine for the prevention and treatment of many ailments in the influential global medical compendium^6^. For example, studies have shown that *Lianhua Qingwen* is more effective than modern therapies in the treatment of COVID-19^7,8^. As an important component of TCM treatment, Chinese patent medicine instructions (CPMI) have significant implications for enhancing the clinical application value of Chinese herbs, standardizing the use of Chinese medicine, and ensuring patient safety.

Currently, research on pre-trained language models (PLMs) in the domain of TCM mainly focuses on entity recognition, clinical record classification, and feature extraction^9–11^. Pan et al.^12^ conducted an in-depth study on electronic medical records (EMRs) in TCM and proposed a named entity recognition (NER) pipeline called the ALBERT-BiLSTM-CRF, which focuses on TCM orthopedic EMRs. The pipeline utilizes the ALBERT as the base model to encode and embed the labeled data, applies BiLSTM to establish comprehensive contextual semantics, and feeds the concatenated vectors into the CRF layer for decoding using the Viterbi algorithm. Experimental results demonstrate that compared to NER models based on BERT, ALBERT-BiLSTM-CRF achieves higher accuracy. To achieve Chinese sentence classification in clinical medical records, Zou et al.^13^ proposed a domain-adaptive PLM called CEMR-LM. CEMR-LM is pre-trained using a large amount of unlabeled clinical corpus to acquire knowledge in the field of TCM. The model's performance is enhanced by combining fine-tuning strategy and a dual-channel mechanism. Chen et al.^14^ conducted a study on coronary heart disease and developed a pre-training diagnostic model based on the BERT model trained on TCM texts. They successfully performed a text classification task on coronary heart disease medical records. The performance improvement compared to the model without TCM pre-training was 0.096. In the feature extraction process in the field of TCM, Chen et al.^15^ combined BERT with a one-dimensional convolutional neural network (1D-CNN) for fine-tuning the pre-trained model. Their model demonstrated a significant improvement in F1 score compared to the traditional 1D-CNN classifier, achieving state-of-the-art performance. Gao et al.^16^ proposed TCM2Vec, which initializes the relationship features between herbs by constructing two independent encoders. One encoder utilizes the unsupervised pre-training model FMh2v based on cross-features, while the other simulates the multi-dimensional features of drugs using a normal distribution. Finally, the relationship and drug features are integrated for deep feature extraction. TCM2Vec serves as an effective method for obtaining feature embeddings of TCM prescriptions, providing crucial insights for the adaptability of artificial intelligence technology in the field of TCM. Overall, research on deep learning in TCM primarily focuses on utilizing structured data and domain knowledge to enhance the automation and analysis efficiency of TCM information processing.

In recent years, researchers have made significant efforts in the field of TCM, paving the way for the development of domain-specific large language models (LLMs) with extensive knowledge in TCM. Wang et al.^17^ conducted supervised fine-tuning of the LLaMA-based model using over 8000 instruction-based question–answer data to develop HuaTuo, aiming to obtain more reliable medical knowledge in the domain of Chinese medicine. Xu et al.^18^ employed a dataset comprising 20,000 TCM records to train an auxiliary diagnostic model based on BERT. The objective of their study was to effectively utilize the information from the four diagnostic methods of TCM and provide TCM-based disease diagnosis for patients. In the study by Zhong et al.^19^, they incorporated semantic features related to TCM acupoints into the corpus and improved BERT through fine-tuning, resulting in a classification model named Bert-Chinese-Acupoint. This model aims to recommend the optimal primary acupoints for treating diseases and diagnose diseases through a classification task. The advantage of domain-specific LLMs over general models lies in their focus and domain expertise. Domain-specific models typically undergo more in-depth learning and understanding of the knowledge within that specific domain, enabling them to provide highly perceptive responses and solutions. This is particularly evident in the domains of TCM consultation, automatic generation of TCM prescriptions, and recommendations for Chinese patent medicine (CPM).

TCM diagnosis is an empirical medical method. TCM practitioners utilize *observation*, *auscultation*, *inquiry and pulse diagnosis*, combined with their personal clinical experience, to comprehensively analyze and diagnose patients^20,21^. However, during this process, subjective judgments and individual experiences of doctors may introduce potential errors, thereby escalating the risk of medical incidents. The utilization of advanced LLMs can mitigate the influence of subjective factors on medical decision-making, enabling clinicians to make precise, patient-centered determinations^22^. As a result, this integration can enhance the overall efficacy and quality of healthcare. Furthermore, the reasoning capabilities inherent in LLMs aligns harmoniously with the fundamental principles of TCM's *synthesis of the four diagnostic methods*, presenting novel avenues and methodologies for TCM research.

Differentiating from previous studies, we present a novel approach for constructing LLMs in the field of CPMI. This approach primarily focuses on automatically generating corresponding recommendations for CPM and detailed instructions for usage based on patients' symptoms and complaints. We constructed an instruction dataset using 3,906 labeled consultation records related to CPM treatments. The foundation model, ChatGLM-6B, was trained with parameter-efficient fine-tuning (PEFT) methods, leading to the development of a novel large-scale language model, CPMI-ChatGLM, specific to the domain of TCM. Evaluating CPMI-ChatGLM involved a combination of automatic assessment and human evaluation, employing metrics such as BELU, ROUGE, BARTScore, and SUS score. By constructing a comprehensive dataset and employing PLMs for the analysis and processing of CPMI, we can identify information such as the drug names, ingredients, specifications, usage, and precautions of TCM. The objective of this study is to assist doctors and patients in gaining a better understanding of the efficacy, dosage, and administration of Chinese medicines, thereby enhancing medication safety, alleviating healthcare burdens, and promoting the inheritance and development of Chinese traditional culture. We have released the dataset of CPMI in the Github repository https://github.com/liucann/CPMI-ChatGLM.

In summary, the main contributions can be summarized as follows:

- To the best of our knowledge, this is the first comprehensive study of CPMI using LLMs. In this study, we proposed a domain-specific language model named CPMI-ChatGLM, which is the first LLM designed for the CPMI.
- We performed instruct-tuning on the foundation model using a high-quality dataset of CPMI.
- We constructed and publicly released the first dataset of CPMI, containing 7 medical specialties and 3906 medical records. Our intention is for this dataset to serve as a valuable resource for both research and application in the field of Chinese medicines.

## Results

### LoRA versus P-Tuning v2

**Figure 1:**
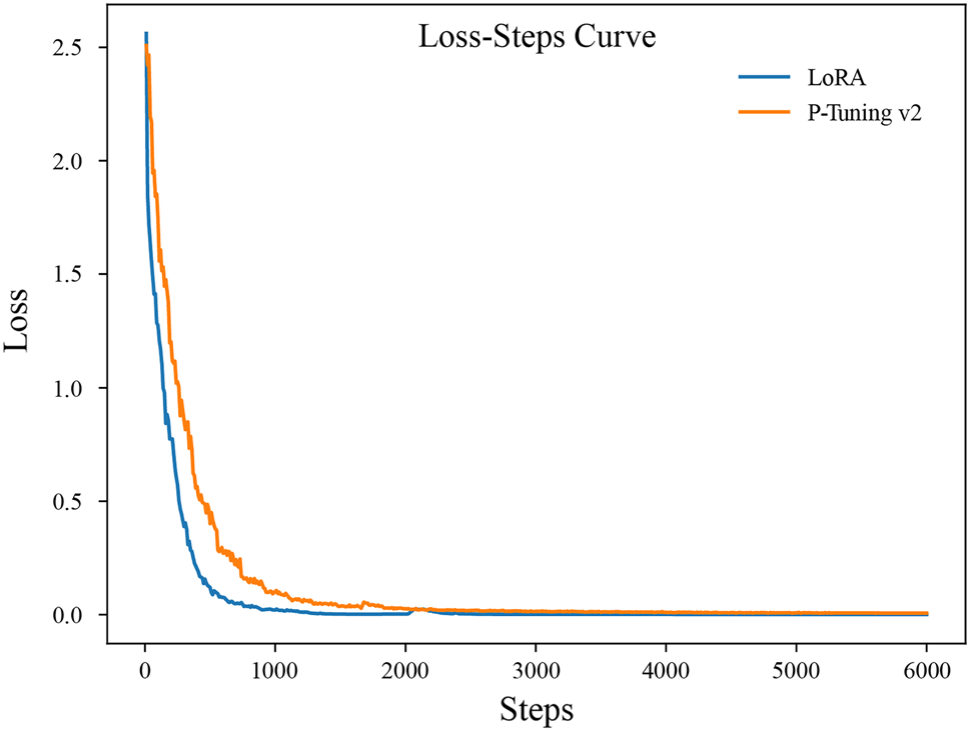
The loss curve of the CPMI-ChatGLM training process. The blue line represents LoRA, while the orange line represents P-Tuning v2.

**Figure 2:**
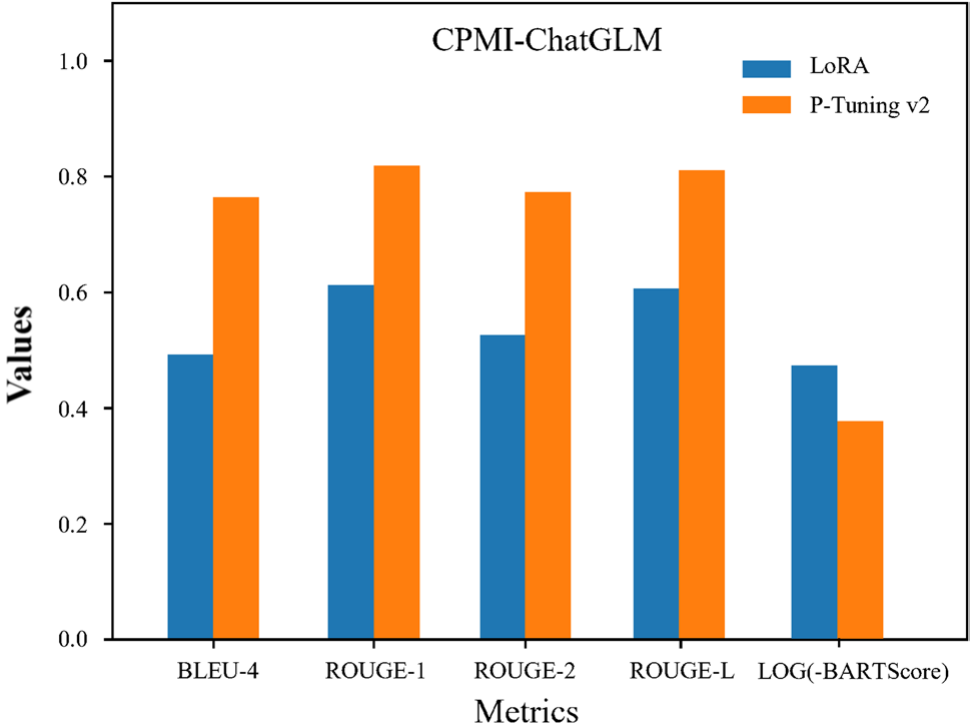
The impact of two fine-tuning methods on the performance of the CPMI-ChatGLM model. It is evident that the P-Tuning v2 fine-tuning method performs better in terms of F1 score. The legend uses blue to represent the LoRA method and orange to represent the P-Tuning v2.

We evaluated two PEFT methods on a server equipped with two RTX 3090Ti (24G) GPUs and assessed their impact on the performance of CPMI-ChatGLM. We used the Rouge_chinese^23^ and NLTK^24^ toolkits to calculate ROUGE and BLEU scores respectively, and BARTScore calculation was performed using the default parameter configuration. Figure 1 shows the training loss curves of the two PEFT methods, and the models converged at around 3000 steps. The smooth loss curves indicate stable performance improvement and effective training. Furthermore, as shown in Fig. 2, CPMI-ChatGLM fine-tuned using the P-Tuning v2 method outperformed LoRA in all aspects. Table 1 presents the specific numerical comparison results for different fine-tuning methods. The results indicate that P-Tuning v2 outperformed LoRA in terms of F1 scores, with improvements of 33.81% for ROUGE-1, 46.93% for ROUGE-2, 33.53% for ROUGE-L, and 55.09% for BLEU. The BARTScore value of P-Tuning v2 was also significantly better than that of the LoRA method. These results have astonished us.

**Table 1:** The evaluation results of two different PEFT methods.

Methods	ROUGE-1			ROUGE-2			ROUGE-L			BLEU-4	BARTScore
	p	r	f	p	r	f	p	r	f		
LoRA	0.6775	0.5741	0.6117	0.5761	0.4997	0.5264	0.6776	0.5690	0.6071	0.4927	− 3.0647
P-Tuning v2	**0.8285**	**0.8175**	**0.8188**	**0.7822**	**0.7730**	**0.7738**	**0.8214**	**0.8102**	**0.8107**	**0.7641**	**− 2.4786**

Compared to the LoRA method, the CPMI-ChatGLM model fine-tuned using P-Tuning v2 demonstrates superior performance. The columns ‘p’ represent precision, ‘r’ stands for recall, and 'f' denotes the F1 score. Bold values indicate superior performance.

We randomly consulted a common issue of gum swelling and mouth ulcers caused by excessive internal heat, and sought recommendations from CPMI-ChatGLM for Chinese patent medicine. The suggestions provided by LoRA were *San Huang Tang*, while P-Tuning v2 recommended the use of *Huanglian Shangqing Wan*. These recommendations were evaluated by clinical TCM practitioner.

The TCM practitioner pointed out that *San Huang Tang* belongs to the category of traditional Chinese medicine formula, and the version commonly available in the market is known as *San Huang Pian* (tablets). *San Huang Pian* is primarily used to address the condition of excessive internal heat in the upper, middle, and lower parts of the body, and it is suitable for symptoms such as gingival swelling, sore throat, and constipation. On the other hand, *Huanglian Shangqing Wan* are mainly used to alleviate oral heat-related symptoms, such as mouth ulcers and gingival swelling. Since the patient only mentioned the presence of oral manifestations of excessive internal heat and did not indicate the existence of such symptoms in other parts of the body, *Huanglian Shangqing Wan* are considered more suitable in this case.

We also discovered that the manufacturer information provided by LoRA was fabricated. The intention behind including information about the drug manufacturer during our training process was to account for potential variations in specifications and dosages of CPM due to different manufacturers. Patients may rely on the manufacturer information to assist them in determining which specification of the medication to purchase and take. The two recommendations provided by CPMI-ChatGLM, as mentioned above, are listed in Table 2.

**Table 2:** Comparison of two fine-tuning methods on a real case.

Input: My husband has been feeling a bit out of balance lately. He's been having toothaches for several days, and there are also some mouth ulcers bothering him. We've been trying some cooling herbal tea to reduce the heat in his body, but there hasn't been any noticeable improvement. Do you have any recommendations for Chinese patent medicines?
LoRA output: It is recommended to take San Huang Tang. Here is the detailed information of it: [Ingredients] Da Huang, Huang Qu, Huang Qin, Huang Bai, Cang Zhu. [Description] This product is a yellow to brownish-yellow decoction with a fragrant aroma, a sweet taste, and a slight bitterness. [Specification] 300 ml/bottle. [Indications] Used for internal heat and heat-related symptoms such as thirst, dry throat, toothache, red complexion, and angular cheilitis. [Usage and Dosage] Children: 6–12 spoonfuls. Adults: 20–30 spoonfuls. Take twice a day, mixing with warm water to form a paste. [Adverse Reactions] Not yet clear. [Manufacturer] Jiguonanding Pharmaceutical Co., Ltd. [Precautions] 1. Avoid spicy and greasy food while taking this product. 2. Pregnant women and diabetes patients should take this product under the guidance of a physician. 3. This product should be taken after meals. 4. It is not suitable for cold and fever patients. 5. People with allergies should use this product with caution. 6. Do not use this product if its characteristics have changed. 7. Do not use this product if its characteristics have changed. 8. Please keep this product out of reach of children. 9. If you are taking other medications, please consult a physician or pharmacist before using this product
P-Tuning v2 output: Huanglian Shangqing Wan may be suitable for treating your condition. Here is the detailed information of it: [Ingredients] Huang Lian, Zhi Zi, Lian Qiao, Man Jing Zi, Fang Feng, Jing Jie Sui, Bai Zhi, Huang Qin, Ju Hua, Bo He, Chi Shao, Dang Gui, Chuan Qiong, Bai Shao, Jie Geng, Gan Cao. [Description] This product is a brownish concentrated pill. It has a bitter taste and has the function of clearing heat. [Specification] 6 g*10 pills. [Indications] Used for clearing heat, dispelling wind, and relieving pain. It is used for symptoms such as dizziness, sore throat, mouth ulcers, nasal congestion, runny nose, headache, and chest pain caused by wind-heat invading the upper body. [Usage and Dosage] Oral administration. Take 1–2 pills per dose, 2–3 times a day. [Adverse Reactions] Not yet clear. [Manufacturer] Tongrentang Pharmaceutical Factory, Beijing Tongrentang Group Co., Ltd. [Precautions] 1. Avoid smoking, alcohol, and spicy foods. 2. It is not advisable to take tonics or nourishing herbs while taking this medication. 3. Patients with chronic diseases such as hypertension, heart disease, liver disease, diabetes, and kidney disease should take this medication under the guidance of a physician. 4. If symptoms do not improve after three days of medication, seek medical attention. 5. Children, elderly, weak, and those with weak spleen and loose stools should take this medication under the guidance of a physician. 6. Strictly follow the recommended dosage and usage. This product is not suitable for long-term use. 7. If the frequency of bowel movements increases and the stool becomes loose after taking the medication, it should be discontinued. Wishing you good health!

### How much data is good enough?

**Figure 3:**
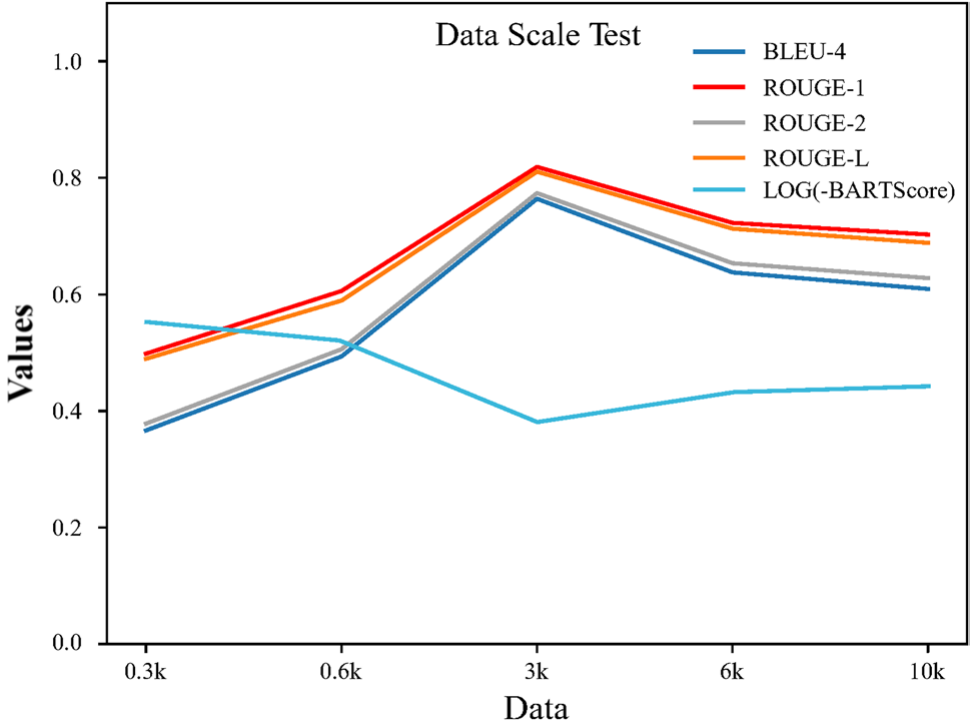
The line chart of various data scales on the performance of CPMI-ChatGLM. The optimal performance of CPMI-ChatGLM is achieved when the data volume reaches approximately 3 k. BLEU and ROUGE employ the F1 score for calculating the scores.

We investigate the influence of dataset size on the performance of CPMI-ChatGLM across five different data scales. These data scales encompass 268 CPM data sourced from the *Guidelines* (0.3 k), a combined dataset of 694 CPMI data collected and meticulously processed from the *Guidelines*, *Tianchi*, and the outpatient department of the Chinese Medicine Hospital (0.6 k), along with 3 k, 6 k, and 10 k data augmented using the Self-chatting method based on the 0.6 k data. The comparison of results from fine-tuning CPMI-ChatGLM with different data scales is shown in Fig. 3.

In general, the performance of a model tends to improve with an increase in data volume^25–27^. This is because larger-scale data provides more information, helping the model better learn features and patterns while reducing the risk of overfitting. Our research findings show that with the inclusion of CPMI data, the performance of CPMI-ChatGLM keeps improving until reaching approximately 3 k data points, as the model learns more data patterns and features. However, as the training data continues to increase, the model starts to overfit the training data, resulting in a decline in performance due to decreased generalization ability on new data.

The answers to the same three problems regarding the recommendation of CPM by different models are provided in the Supplementary Table S1. Specifically, the model that has not undergone fine-tuning, known as the foundation model ChatGLM-6B, exhibits limited capability in recommending CPM. It simply suggests some suitable names of CPM without providing specific instructions on administration methods and precautions. After fine-tuning with small-scale datasets, specifically datasets consisting of 0.3 k and 0.6 k, the CPMI-ChatGLM model's answers mostly adhere to the required format. However, its ability to accurately understand the symptoms and recommend CPM with corresponding efficacy still requires improvement. Conversely, as the dataset reaches a certain scale, specifically 6 k and 10 k, the model's generated unrealistic outcomes gradually increase, resulting in the inclusion of Chinese medicine names that do not exist in reality and even incorrect medication precautions in the answers.

### Ablation study of instruction data

By providing specific prompt words, prompt engineering can help the model better understand and generate text, thereby improving its performance and achieving satisfactory results^28^. Similar to prompts, fine-tuning the model using instruction data can enhance the model's adaptability to specific tasks and scenarios, often leading to more prominent performance outcomes.

In this section, we conducted ablation experiments on the instruction dataset of CPMI. By removing the instruction part from the dataset and fine-tuning the foundation model using the same hyperparameters and the P-Tuning v2 method, we compared the performance of instruction tuning with non-instruction tuning. The experimental results demonstrate that instruction tuning can significantly improve the performance of the CPMI-ChatGLM model, enhancing both the accuracy and coherence of the generated text. The experimental results in Table 3 show that instruction tuning improves the performance of CPMI-ChatGLM by approximately 20%. This also indicates that instruction tuning can help large-scale models better understand the context and intent of the input, resulting in more accurate and expected text generation, thereby improving the quality and effectiveness of the model.

**Table 3:** The results of ablation study.

	ROUGE-1	ROUGE-2	ROUGE-L	BLEU-4	BARTScore
Non-instruction data	0.6852	0.6112	0.6726	0.5947	− 2.8207
Instruction data	**0.8188**	**0.7738**	**0.8107**	**0.7641**	**− 2.4786**

Fine-tuning with instruction data achieves better performance than non-instruction tuning. The ROUGE scores in the table are all reported as F1 scores. Bold values indicate better performance.

### Comparisons to other models

To demonstrate the superior performance of CPMI-ChatGLM and ensure the fairness of the experiments, we conducted a comparative analysis by comparing it with four widely-used models that employ Chinese pre-training corpus and possess a similar scale of parameters.

- Chinese-LLaMA-7B^29^: This model is an extension of LLaMA-7B that incorporates Chinese vocabulary and continues pre-training with Chinese embeddings, resulting in a Chinese-specific LLaMA model.
- Chinese-Alpaca-7B^29^: Building upon the Chinese-LLaMA-7B model, the Chinese-Alpaca-7B was further fine-tuned using an instruction dataset, resulting in an improved Chinese LLaMA model.
- Qwen-7B^30^: An open-source model from Alibaba Group Qwen series, with a parameter size of 7 billion. Qwen-7B is a large-scale Transformer-based language model trained on an extensive range of pre-training data, including a diverse collection of web text, professional books, and code.
- Baichuan-7B^31^: Developed by Baichuan Intelligent Technology, Baichuan-7B is an open-source, large-scale pre-training model. With 70 billion parameters trained on approximately 1.2 trillion tokens, this transformer-based model supports both Chinese and English. It achieves the best performance among models of the same size on standard Chinese and English benchmarks (C-EVAL^32^/MMLU^33^).

The comparative experimental results of the models are presented in Table 4. Among models of comparable scale, our CPMI-ChatGLM achieves the best performance. Regarding the composition of the training corpora, Qwen-7B and Baichuan-7B, which undergo pre-training on a larger volume of Chinese text, outperform Chinese-LLaMA-7B and Chinese-Alpaca-7B, which are based on LLaMA-7B pre-trained using English text. Moreover, Chinese-Alpaca-7B exhibits superior performance compared to Chinese-LLaMA-7B, which lacks prompt fine-tuning.

**Table 4:** Comparative experimental results of different models.

Model	ROUGE-1	ROUGE-2	ROUGE-L	BLEU-4	BARTScore
Chinese-LLaMA-7B	0.4680	0.3436	0.4542	0.3098	-3.5239
Chinese-Alpaca-7B	0.5205	0.4095	0.5059	0.3818	-3.4108
Qwen-7B	0.7370	0.6702	0.7261	0.6506	-2.7180
Baichuan-7B	0.7745	0.7176	0.7639	0.6996	-2.6158
CPMI-ChatGLM (Our)	**0.8188**	**0.7738**	**0.8107**	**0.7641**	**-2.4786**

Bold values indicate superior performance.

### Human evaluation

Although automatic metrics play a certain role in evaluating the performance of LLMs, human evaluation remains necessary to ensure the model’s performance in terms of safety, validation of professional knowledge, flexibility and adaptability, as well as ethical considerations. For the Chinese medicine QA task, our study introduces the SUS (Safety, Usability, and Smoothness) human evaluation method^17^. The SUS consists of three dimensions: safety, usability, and smoothness. The “safety” dimension assesses whether the model-generated content has the potential to mislead users and pose a risk to their health. The “usability” dimension evaluates the extent to which the generated content reflects professional knowledge, while the “smoothness” dimension measures the proficiency of the generation model as an LLM. SUS adopts a three-point scoring mechanism, with scores ranging from 1 (unacceptable) to 3 (good), and 2 indicating an acceptable performance. To evaluate the model's performance, we recruited five raters with a background in Chinese medicine to score 20 randomly selected questions regarding CPM recommendations. Table 5 presents the average SUS scores along with their corresponding 95% confidence intervals.

**Table 5:** Table 5. The SUS scores and their corresponding 95% confidence intervals across different models.

	Safety	Usability	Smoothness
Chinese-LLaMA-7B	2.100 ± 0.566	1.910 ± 0.560	2.410 ± 0.574
Chinese-Alpaca-7B	2.187 ± 0.550	1.967 ± 0.550	2.319 ± 0.395
Qwen-7B	2.600 ± 0.424	2.420 ± 0.440	2.710 ± 0.325
Baichuan-7B	2.767 ± 0.243	2.637 ± 0.296	2.827 ± 0.201
CPMI-ChatGLM (Our)	**2.880±0.163**	**2.780±0.314**	**2.950±0.127**

Bold values indicate superior performance.

Compared to other models, Chinese-LLaMA-7B and Chinese-Alpaca-7B generate content that contains a higher number of English letters and symbols, which significantly impacts usability and fluency, leading to a diminished user experience and lower usability scores. On the other hand, our CPMI-ChatGLM model markedly enhances the usability of knowledge while ensuring the security and fluency of the results.

### Model parameter setting and cost

We developed CPMI-ChatGLM by fine-tuning ChatGLM-6B using the PEFT approach. Despite having only 6.2 billion parameters, ChatGLM-6B still performs consistently with human preferences. The detailed hyperparameter information for fine-tuning ChatGLM-6B using the LoRA algorithm and P-Tuning v2, as well as the specific hyperparameter details for other comparative models, are provided below:

For LoRA fine-tuning, we conducted fine-tuning for 5 h on two RTX 3090Ti (24 GB) GPUs. The batch size during training was set to 2, the learning rate for the AdamW^34^ optimizer was set to 2e−5, the total number of training steps was 6000, and the maximum sequence length was set to 256. No specific settings were applied for warmup and weight decay. Additionally, the rank of the low-rank matrix was set to 8, the scaling factor was set to 32, and the dropout rate was set to 0.1.

For P-Tuning v2, the batch size was set to 1. We used the AdamW optimizer with the default learning rate decay strategy, setting the learning rate to 2e−2. The gradient accumulation was performed every 16 steps, the maximum source length was set to 32, and the maximum target length was set to 256. The total number of training steps, similar to LoRA, was set to 6000, and no warmup or weight decay was applied. Under 4-bit quantization, CPMI-ChatGLM achieved inference on a single RTX 3090Ti (24 GB) GPU in just 5 h, consuming approximately 8 GB of GPU memory, making it affordable for most researchers.

Four comparison models were trained using supervised fine-tuning, with a maximum input sequence length of 512. For Chinese-LLaMA-7B and Chinese-Alpaca-7B, the batch size for training was set to 4. The AdamW optimizer was employed with an initial learning rate of 1e-4. Both models employed the constant warmup method as the learning rate scheduling strategy, dynamically adjusting the learning rate. Gradient accumulation was performed every 4 steps, and both models were trained for 10 epochs. For Qwen-7B, a batch size of 4 was used for training. The AdamW optimizer had an initial learning rate of 1e-3, and the learning rate scheduling was conducted using the cosine annealing strategy. The number of gradient accumulations was 4, and a total of 8 epochs were trained. For Baichuan-7B, the batch size for training was set to 8. The initial learning rate was 1e−4, and the learning rate was dynamically adjusted using the cosine annealing strategy. Gradient accumulation was performed every 4 steps, and a total of 10 epochs were trained.

## Discussion

Currently, large-scale models in the medical field primarily focus on extracting symptoms and clinical entities from the input to ensure the generation of healthcare recommendations^35–37^. While this approach is beneficial for diagnosing and treating diseases, there is still a lack of emphasis on generating detailed usage instructions for medications. In reality, patients are more interested in directly understanding the specific usage instructions and dosages for each administration, as this information is crucial for the proper use, therapeutic efficacy, and safety of medications.

We collected and constructed labeled CPM data through three sources: *Standard Therapeutic Guidelines for National Essential Drugs (Chinese Patent Medicine)*, *Entity Recognition of Traditional Chinese Medicine*, and hospital consultation records. This was done to address the need for CPM recommendations and detailed usage instructions. Large-scale models are capable of identifying and analyzing various aspects of medications, including their ingredients, dosages, administration methods, and precautions. Leveraging the advantages of PLMs, we have implemented specific CPM recommendations and provided detailed usage instructions. This enables doctors and patients to access more comprehensive and accurate information about medications, helping them better understand the effects and possible side effects of the medications. Furthermore, the model can offer personalized medication advice based on the specific conditions of patients, ensuring the safety and effectiveness of the medications.

We fine-tuned the base model ChatGLM-6B to develop the CPMI-ChatGLM model for generating CPMI. By using the PEFT method to study the model's performance, we found that the model fine-tuned with P-Tuning v2 outperformed LoRA in all evaluation metrics. Regarding data scale, the performance of CPMI-ChatGLM reached its peak at a data volume of 3 k and declined with further increases. This trend may be attributed to the diversity of the data. In addition, we also investigated the influence of instruction data on model performance and conducted a comparative analysis among various commonly employed LLMs. Through a comprehensive evaluation combining both automatic metrics and human evaluation, we demonstrated the superiority of CPMI-ChatGLM. Considering resource and cost limitations, we selected a set of 3096 meticulously labeled data for fine-tuning CPMI-ChatGLM. Ultimately, we completed the 4-bit quantization P-Tuning v2 fine-tuning on a single RTX3090Ti GPU, consuming approximately 8 GB of GPU memory, making it accessible for most researchers to deploy locally. Furthermore, we have made the original dataset used in this study publicly available on our GitHub repository for reference and use by other researchers.

However, this study has certain limitations. Firstly, the relatively small parameter size of the foundation model and the data scale used may result in errors such as the inclusion of English characters in the generated Chinese text. To address this issue, we plan to experiment with larger-scale foundation models and incorporate manual evaluation methods to ensure higher quality. Secondly, we aim to expand the corpus beyond TCM to other categories of drugs, improving model performance that may be affected by data diversity, thereby making the model applicable to a wider range of medical conditions. In terms of model training, we intend to incorporate image-assisted information (such as pictures of the patient's affected area) to enable multimodal medical inquiry, further enhancing diagnostic accuracy and safety.

## Conclusions

In conclusion, we have developed a new large-scale model, CPMI-ChatGLM, in the field of TCM, exploring new avenues for the integration of TCM and artificial intelligence. Through the PEFT method, we achieved superior performance compared to the foundation model by fine-tuning with instruction data. We also investigated the impact of different fine-tuning methods, data scales, and instruction data on the performance of CPMI-ChatGLM. Additionally, we have publicly released the first dataset of CPMI, aiming to contribute to the modernization and internationalization of TCM. Currently, the CPMI-ChatGLM project is in its early stages and may contain errors. We are actively collaborating with hospitals and medical experts to seek feedback and suggestions in order to improve its medical accuracy and assistive capabilities.

## Methods

### Dataset and data preprocessing

Our original dataset primarily derives from *Standard Therapeutic Guidelines for National Essential Drugs (Chinese Patent Medicine)* (ISBN 9787117286916). The guideline provides comprehensive and systematic information on 268 CPMs across 7 specialized fields, including internal medicine, surgery, gynecology, ophthalmology, otolaryngology, orthopedics, and pediatrics. It serves as a valuable resource, offering extensive and well-organized information on CPM. To enhance the model's capacity to learn various disease types, we expanded our dataset by merging it with *Entity Recognition of Traditional Chinese Medicine's Manual* from Aliyun Tianchi^38^. The Tianchi dataset comprises 1997 records sourced from instructions for TCM. It encompasses 13 key categories, including drugs, drug components, syndromes, properties, flavors, and Chinese medicinal effects. By incorporating this dataset into our corpus, the model can benefit from diverse sources of information, thereby improving its recognition and learning capabilities and enhancing overall performance. Additionally, we collected a set of 100 patient consultation records and corresponding CPM prescriptions from our affiliated TCM hospital outpatient department. After removing personal information, these additional data were merged into the corpus. The inclusion of these data supplements allows for a more accurate reflection of the actual application of CPM and further enhances the accuracy of the dataset.

We extracted information on the drug name, ingredients, description, specifications, indications, usage and dosage, adverse reactions, and precautions of CPM from 7 specialized medical fields in the *Guidelines* and manually added information on the manufacturer to construct a dataset of CPMI. However, some drug instructions in the Tianchi dataset had inconsistent attributes, such as the absence of a “drug name” or “indications” attribute, which are crucial for understanding the drug usage rules. To prevent the model from experiencing hallucinatory effects that could lead to incorrect guidance for users, we removed drugs with unknown attributes, retaining only 326 records without unknown labels.

Inspired by the “Self-chatting” approach in Baize^39^, we employed ChatGLM for data augmentation of our processed dataset. As a result, we generated five additional sentences that maintain a similar meaning and intent to the original patient's complaints in the dataset. These new sentences were then added to the dataset, resulting in a six-fold expansion of the dataset. In addition, to ensure the rationality and safety of CPM usage, we invited two senior clinical practitioners of TCM with advanced professions to review the CPMI dataset, further enhancing the quality and effectiveness of the dataset. After eliminating 258 instances of false and erroneous information caused by the hallucinations of LLMs, the dataset contains a total of 3906 data records.

### Foundation model

ChatGLM-6B is an open-source conversational language model that supports bilingual question-answering in Chinese and English. It adopts the same model architecture as GLM-130B^40^ and utilizes the general language model (GLM) as its backbone. GLM^41^ is a transformer-based language model trained with autoregressive blank filling as the objective, supporting INT4 quantization and efficient inference on a single RTX 3090 GPU. In a comprehensive evaluation of 30 leading large models worldwide conducted by the Stanford Institute for Human-Centered Artificial Intelligence (HAI), GLM-130B was the only selected model from Asia^42^. In the holistic evaluation conducted at HAI, 16 core scenarios were evaluated, encompassing tasks such as question answering, information retrieval, summarization, sentiment analysis, and more. The evaluation included seven metrics: accuracy, calibration, robustness, fairness, bias, toxicity, and efficiency. This comprehensive approach ensures that indicators beyond accuracy receive due attention and explicitly highlights the trade-offs between models and metrics. Furthermore, a targeted assessment was carried out for 26 specific scenarios to delve deeper into specific aspects, such as knowledge, reasoning, memory/copyright, and misinformation. Among the 16 common task scenarios, GLM-130B exhibited outstanding performance in text classification tasks, achieving an impressive overall accuracy rate of 85.8%. In the evaluation of the seven metrics, GLM-130B demonstrated remarkable performance in terms of accuracy, fairness, toxicity, and overall text generation bias, reaching a level comparable to GPT-3 davinci v1 (175B). Additionally, GLM-130B showcased better robustness, calibration error, and lack of bias compared to GPT-3 in general.

ChatGLM-6B adopts a prefix decoder-only transformer framework, incorporating bidirectional attention mechanism for input and unidirectional attention mechanism for output. In terms of model details, ChatGLM-6B employs gradient scaling for the embedding layer and utilizes Post-LN layer normalization method to enhance training stability. Additionally, rotary positional encoding (RoPE) is employed as a replacement for traditional absolute positional encoding, and GeLU activation is used to improve the feedforward networks (FFNs) in the transformer architecture. Here, we adopted the ChatGLM-6B for more convenient training.

### Our CPMI-ChatGLM

To transferring the knowledge from general domain to the CPM, we performed fine-tuning of the ChatGLM-6B on a dataset containing 3906 labeled CPMIs that we constructed. The resulting model, CPMI-ChatGLM, serves as an automated CPMI generator, providing prescribing recommendations for physicians and patients.

**Figure 4:**
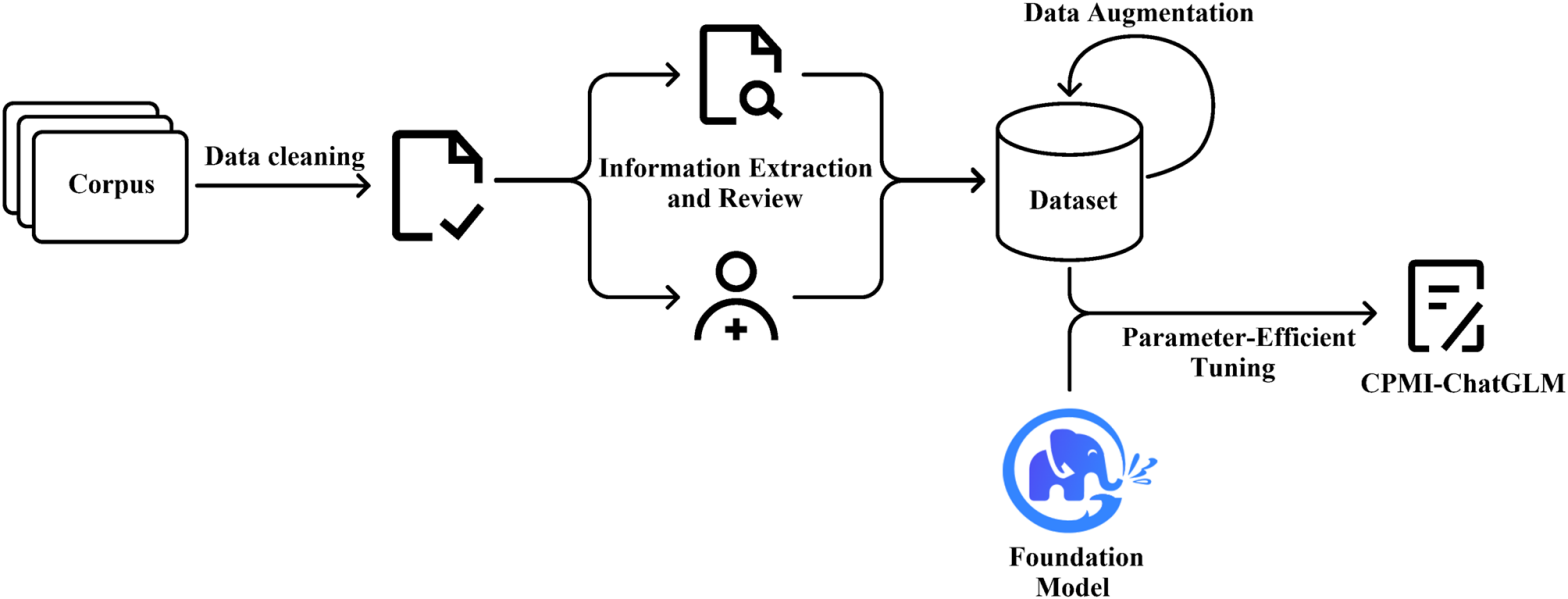
The pipeline for training CPMI-ChatGLM. The collected corpus of CPMI was subjected to preliminary data cleaning, followed by machine screening and TCM expert review to form the training dataset. Then the ChatGLM was used to expand the dataset, and the foundation model was parameter-efficient fine-tuned to construct CPMI-ChatGLM, an LLM specifically designed for traditional Chinese medicine instructions.

Figure 4 illustrates the workflow for constructing the CPMI-ChatGLM model for CPMI generation. Firstly, we collected data from various sources and constructed the CPMI corpus. After the initial data cleaning, which involved removing special characters and adjusting the format, the data underwent extraction of key attributes and a thorough review process. This step included the assessment of information by both machine algorithms and experts in TCM. The extracted key attributes were then used to form the final dataset, ensuring both accuracy and security. Subsequently, we employed ChatGLM for data augmentation, further enhancing the dataset size to improve the model’s performance. Finally, we fine-tuned the foundation model, ChatGLM-6B, using the PEFT method, resulting in the CPMI-ChatGLM model. The primary functionality of the model is to automatically generate recommendations for CPM treatment and corresponding detailed instruction information based on user-provided symptoms. This capability holds potential in assisting physicians with diagnosis and improving patient visit efficiency.

### Parameter-efficient fine-tuning

With the rise of LLMs, the parameter of PLMs has increased dramatically^43^. However, due to resource and cost limitations, it has become impractical for ordinary researchers to perform full-parameter fine-tuning of LLMs on consumer-grade hardware. Additionally, storing and deploying separate fine-tuned models for each downstream task has become prohibitively expensive, as the size of the fine-tuned models remains the same as the original pre-trained model. To address these challenges, PEFT was proposed^44^. PEFT method involves fine-tuning only a small or additional set of model parameters while keeping the majority of pre-training parameters fixed, resulting in significant reductions in computation and storage costs. Moreover, advanced PEFT techniques such as Adapter-Tuning, Prefix-Tuning, P-Tuning, and LoRA have achieved performance comparable to full fine-tuning.

Adapter-Tuning^45^ involves inserting smaller neural network layers or modules (referred to as adapters) into each layer of the pre-trained model. During the fine-tuning process, the parameters of the original transformer are frozen, and only the parameters of the adapter layers are learned. Prefix-Tuning^46^ adds additional trainable prefix pseudo-tokens to the input or hidden layers of the model, and only these prefix parameters are trained.

**Figure 5:**
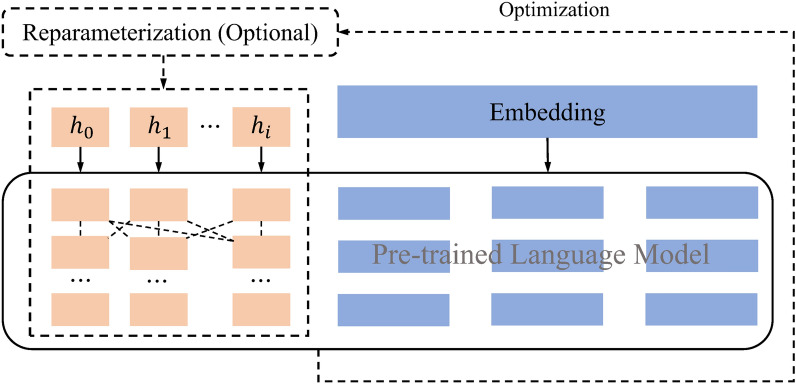
The schematic diagram of P-Tuning v2. The orange blocks (i.e., ${h}_{0}$,…,${h}_{i}$) represent trainable prompt embeddings, and the blue part represents the embedding layer, which is stored or computed by a frozen pre-trained language model. The dashed arrow that returns to the input indicates the optional reparameterization optimization mode.

P-Tuning^47^ follows a similar approach to Prefix-Tuning by utilizing a small number of continuous embedding parameters as prompts to improve the application of generative pre-trained transformer (GPT) in natural language understanding (NLU) tasks. The difference lies in the fact that Prefix-Tuning is designed for natural language generation (NLG) tasks, whereas P-Tuning focuses on adding parameters only in the embedding layer, as opposed to introducing trainable parameters in every layer like Prefix-Tuning. P-Tuning v2^48^ applies Prefix-Tuning to NLU tasks, specifically the orange section in Fig. 5, and applies continuous prompts at each layer of the model while optimizing the prompt parameters.

**Figure 6:**
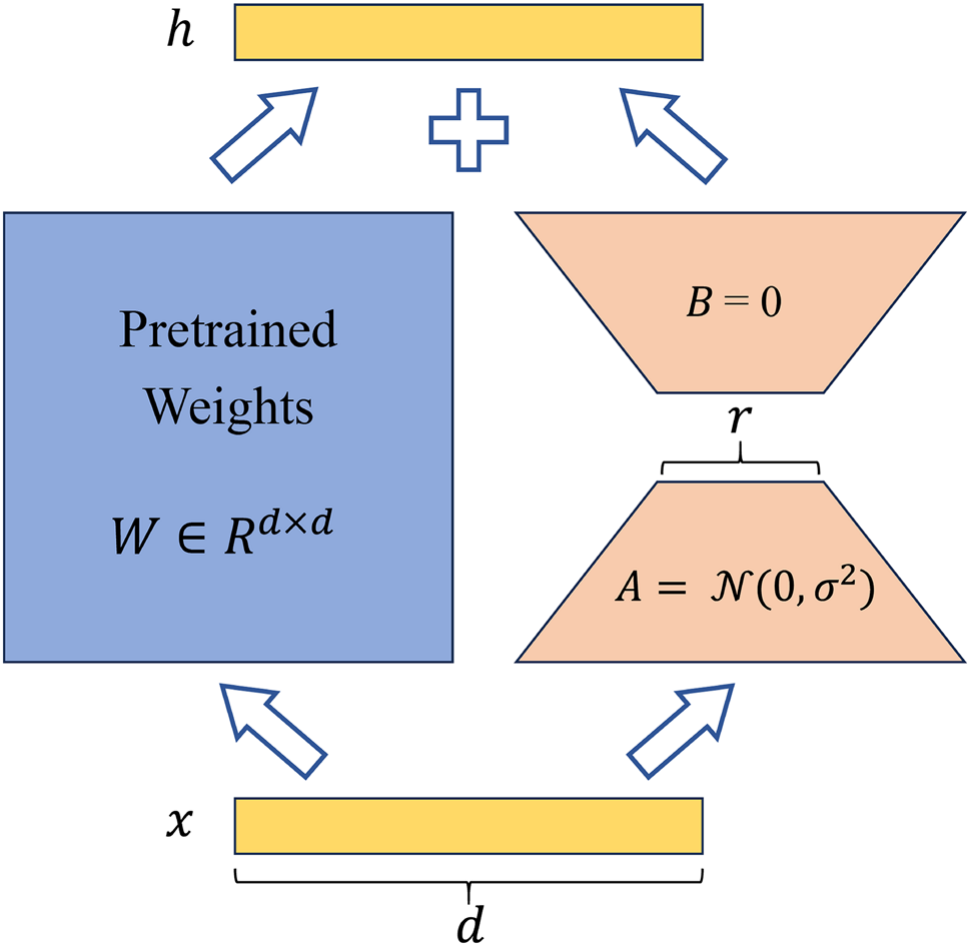
The forward propagation of data in LoRA. The input data $x$ is fed into a weight matrix $W$ on the left and two weight matrices $A$ and $B$ on the right. The hidden layer output dimensions of both sides are equal, with a value of $d$. The output results from the left and right sides are combined through addition to yield the final output result, denoted as $h$.

LoRA^49^ simulates full-parameter fine-tuning by introducing auxiliary matrices $A$ and $B$. It approximates the parameter updates of the model's weight matrix using low-rank matrices learned from small parameters. During training, only the parameters of the low-rank matrices are optimized. For the linear layer $h=Wx$, the forward propagation is replaced with the following formulation:

1 $$\begin{array}{c}h=Wx+BAx\end{array}$$

where $W\in {R}^{d\times d}$, $A\in {R}^{d\times r}$, $B\in {R}^{r\times d}$, with the rank $r\ll d$, Matrix $A$ is initialized with a random Gaussian distribution, while matrix $B$ is initialized with all zeros, ensuring that only the main branch is active during the initial stage. The forward propagation of data in LoRA is illustrated in Fig. 6.

In this study, we employed the P-Tuning v2 method to fine-tune the ChatGLM-6B model and compared it with the LoRA fine-tuning method using the same instruction data. The aim was to develop a high-performing, cost-effective, and applicable language model for the field of TCM to meet practical application needs.

### PEFT with instruction data

Fine-tuning LLMs using machine-generated instruction data enables significant zero-shot capabilities on new tasks without the need for manual instruction writing^50^. Inspired by Self-Instruct^51^, data can be self-bootstrapped to enhance the ability of PLMs to follow instructions. By incorporating TCM knowledge, we provide instructions to guide the model in correctly answering with appropriate CPM for input medical cases. For domain-specific tasks, compared to general-domain models, it is often sufficient to use a small set of instructions to guide data generation. This strategy improves model performance in specific domain tasks while reducing the preparation and processing costs associated with instruction data, thus enhancing the efficiency of model training. Table 6 displays the instruction data utilized in this study to guide the generation of CPMI.

**Table 6:** An example of instruction data.

Instruction: Applying traditional Chinese medicine knowledge to provide accurate answers with appropriate Chinese patent medicine for this particular case
Input: I’ve been experiencing a really dry throat, and it’s painful when I swallow saliva, almost like a burning sensation
Output: It is recommended to take Fufang Donglingcao Hanpian. The following is the detailed information of Fufang Donglingcao Hanpian…

### Metrics

In this study, Bilingual Evaluation Understudy (BLEU)^52^, Recall-Oriented Understudy for Gisting Evaluation (ROUGE)^23^ and BARTScore^53^ are employed to evaluate the degree of match between the candidate text and the reference text. These metrics enable us to comprehensively assess the performance of the model in terms of accuracy, fluency, and information completeness, thereby providing guidance for the improvement and optimization of the model.

BLEU is a metric used to measure the similarity between two texts. It is calculated using the following formula:

2 $$\begin{array}{c}BLEU=BP*{\text{exp}}\left(\frac{1}{n}\sum_{i=1}^{N}{P}_{n}\right)\end{array}$$

$BP$ in BLEU stands for brevity penalty, which penalizes excessively short sentences to prevent the model from favoring shorter sentences during training. The expression for calculating the $BP$ is as follows:

3 $$BP = \left\{ {\begin{array}{*{20}c} {1,} & {if\;l_{c} > l_{s} } \\ {e^{{1 - \frac{{l_{s} }}{{l_{c} }}}} ,} & { if l_{c} \le l_{s} } \\ \end{array} } \right.$$

In Formula 3, ${l}_{c}$ represents the length of the candidate, and ${l}_{s}$ represents the effective length of the reference. When the length of the candidate is greater than the length of the reference, the $BP$ is 1, indicating no penalty. Otherwise, the $BP$ is calculated.

In addition, in Formula 2, ${P}_{n}$ represents the precision based on n-grams, which is expressed as follows:

4 $$\begin{array}{c}{P}_{n}= \frac{\sum_{i}^{E}\sum_{k}^{K}{\text{min}}\left({h}_{k}\left({c}_{i}\right),{ \, min}_{j\in M}{h}_{k}({s}_{i,j})\right) }{\sum_{i}^{E}\sum_{k}^{K}{\text{min}}\left({h}_{k}\left({c}_{i}\right)\right)}\end{array}$$

$E$ represents the total number of candidate texts, $K$ represents the total number of word groups. ${h}_{k}\left({c}_{i}\right)$ represents the frequency of the $k$ th word group appearing in the candidate text ${c}_{i}$. ${s}_{j}$ represents the reference, where $j\in M$, and $M$ represents the number of reference answers. ${h}_{k}({s}_{i,j})$ represents the frequency of the $k$ th word group appearing in the standard answer ${s}_{i,j}$.

ROUGE is one of the commonly used metrics in the field of text summarization for evaluating the quality of automatically generated summaries. It measures the overlap between the basic units of the summaries generated by a statistical model and the reference summaries created by humans. The formula for calculating ROUGE is as follows:

$$ROUGE - N = \frac{{\mathop \sum \nolimits_{{S \in \left\{ {Reference\;Summaries} \right\}}} \mathop \sum \nolimits_{{gram_{n} \in S}} Count_{match} \left( {gram_{n} } \right)}}{{\mathop \sum \nolimits_{{S \in \left\{ {Reference\;Summaries} \right\}}} \mathop \sum \nolimits_{{gram_{n} \in S}} Count\left( {gram_{n} } \right)}}$$

5 $$\begin{array}{c}. \end{array}$$

In Formula (5), $n$ represents the length of n-grams, and ${Count}_{match}\left({gram}_{n}\right)$ denotes the maximum number of occurrences of n-grams that appear simultaneously in the candidate text and the reference text. ROUGE-1 measures the matching of unigrams, ROUGE-2 measures the matching of bigrams, and ROUGE-L captures the longest common subsequence, and so on.

BARTScore is the state-of-the-art metric proposed by Yuan et al. for evaluating natural language generation (NLG) in a general context. The concept behind BARTScore involves assessing the quality of sentences based on the generation probabilities derived from the large-scale pre-trained model BART^54^. It computes the logarithmic probability of each token in the hypothesis using an autoregressive approach and then averages them to obtain the overall score. This evaluation process can be formally expressed as:

6 $$\begin{array}{c}BARTSCORE=\sum_{t=1}^{m} \, {\omega }_{t} \, logp\left({{\varvec{y}}}_{t}\mid {{\varvec{y}}}_{<t},{\varvec{x}},\theta \right)\end{array}$$

$\theta$ represents a seq2seq model, consisting of a source sequence containing $n$ tokens $x = \{{x}_{1}, ..., {x}_{n}\}$ and a target sequence containing $m$ tokens $y = \{{y}_{1}, ..., {y}_{m}\}$. The weight of the $t$-th target sequence is denoted as ${\omega }_{t}$.

### Supplementary Information

Supplementary Table S1.

## Data Availability

The public datasets used in this study include the *Standard Therapeutic Guidelines for National Essential Drugs (Chinese Patent Medicine)* with ISBN 9787117286916, as well as *Entity Recognition of Traditional Chinese Medicine* from Aliyun Tianchi https://tianchi.aliyun.com/dataset/86819. The dataset of CPMI in the Github repository https://github.com/liucann/CPMI-ChatGLM.

## References

[1] Du H-Z, Hou X-Y, Miao Y-H, Huang B-S, Liu D-H (2020). Traditional Chinese medicine: an effective treatment for 2019 novel coronavirus pneumonia (NCP). Chin. J. Nat. Med..

[2] Zeng H (2021). History and development of TCM case report in a real-world setting. Evid.-Based Complement. Altern. Med..

[3] Sun Q (2021). Traditional Chinese medicine and colorectal cancer: Implications for drug discovery. Front. Pharmacol..

[4] Luo H (2020). Reflections on treatment of COVID-19 with traditional Chinese medicine. Chin. Med..

[5] Zhu L (2020). The treatment of intervertebral disc degeneration using traditional Chinese medicine. J. Ethnopharmacol..

[6] Cyranoski D (2018). Why Chinese medicine is heading for clinics around the world. Nature.

[7] Liu M (2021). Efficacy and safety of herbal medicine (Lianhuaqingwen) for treating COVID-19: A systematic review and meta-analysis. Integr. Med. Res..

[8] Lee DYW, Li QY, Liu J, Efferth T (2021). Traditional Chinese herbal medicine at the forefront battle against COVID-19: Clinical experience and scientific basis. Phytomedicine.

[9] Zhang T (2022). Information extraction from the text data on traditional Chinese medicine: A review on tasks, challenges, and methods from 2010 to 2021. Evid.-Based Complement. Altern. Med..

[10] Ni P, Okhrati R, Guan S, Chang V (2022). Knowledge graph and deep learning-based text-to-GraphQL model for intelligent medical consultation chatbot. Inf. Syst. Front..

[11] Ahmad PN, Shah AM, Lee K (2023). A review on electronic health record text-mining for biomedical name entity recognition in healthcare domain. Healthcare.

[12] Xuefeng, P., Yuanyuan, C., Xiaorui, H., & Wei, S. Named entity recognition of TCM electronic medical records based on the ALBERT-BiLSTM-CRF model. In *2022 12th International Conference on Information Technology in Medicine and Education (ITME)* 575–582. 10.1109/ITME56794.2022.00125 (2022).

[13] Zou, Y. *et al.* A domain adaptive pre-training language model for sentence classification of Chinese electronic medical record. In *2023 IEEE International Conference on Bioinformatics and Biomedicine (BIBM)* 4776–4783. doi:10.1109/BIBM58861.2023.10386068 (2023).

[14] Chen H, Qin D, Zhang X, Zhang H, Liang X, Liu F, Duan N, Xu Q, Hong Y (2023). Chest Impediment as an Example. Natural Language Processing and Chinese Computing.

[15] Chen, T., Wu, M. & Li, H. A general approach for improving deep learning-based medical relation extraction using a pre-trained model and fine-tuning. *Database: J. Biol. Databases Curation* **2019**, baz116 (2019). 10.1093/database/baz116 PMC6892305 31800044

[16] Gao W, Cheng N, Xin G, Khantong S, Ding C (2023). TCM2Vec: A detached feature extraction deep learning approach of traditional Chinese medicine for formula efficacy prediction. Multimed. Tools Appl..

[17] Wang, H. *et al.* HuaTuo: Tuning LLaMA model with chinese medical knowledge. Preprint at 10.48550/arXiv.2304.06975 (2023).

[18] Xu, C., Yuan, F., & Chen, S. Research on assistant diagnostic method of TCM Based on BERT. In *2021 11th International Conference on Information Technology in Medicine and Education (ITME)* 282–286. 10.1109/ITME53901.2021.00065 (2021).

[19] Zhong X, Jia Y, Li D, Zhang X (2021). Classification of acupuncture points based on the Bert model*. J. Data Anal. Inf. Process..

[20] Yang X (2023). The inheritance of Chinese narrative medicine practice to the philosophical wisdom of traditional Chinese medicine. Chin. Med. Cult..

[21] Niu Y, Hua J, Feng L (2020). Traditional Chinese Medicine Diagnosis and Treatment. Thirty Great Inventions of China: From Millet Agriculture to Artemisinin.

[22] Sezgin E (2023). Artificial intelligence in healthcare: Complementing, not replacing, doctors and healthcare providers. Digital Health.

[23] Lin, C.-Y. ROUGE: A package for automatic evaluation of summaries. In *Text Summarization Branches Out* 74–81 (Association for Computational Linguistics, 2004).

[24] Bird, S. NLTK: The natural language toolkit. In *Proceedings of the COLING/ACL 2006 Interactive Presentation Sessions* 69–72 (Association for Computational Linguistics, 2006). 10.3115/1225403.1225421.

[25] Urbizu, G., San Vicente, I., Saralegi, X. & Corral, A. Not enough data to pre-train your language model? MT to the Rescue! In *Findings of the Association for Computational Linguistics: ACL 2023* 3826–3836 (Association for Computational Linguistics, 2023).

[26] Diao, S. *et al.* Taming pre-trained language models with N-gram representations for low-resource domain adaptation. In *Proceedings of the 59th Annual Meeting of the Association for Computational Linguistics and the 11th International Joint Conference on Natural Language Processing (Volume 1: Long Papers)* 3336–3349 (Association for Computational Linguistics, Online, 2021). 10.18653/v1/2021.acl-long.259.

[27] Edwards, A., Camacho-Collados, J., De Ribaupierre, H. & Preece, A. Go simple and pre-train on domain-specific corpora: On the role of training data for text classification. In *Proceedings of the 28th International Conference on Computational Linguistics* 5522–5529 (International Committee on Computational Linguistics, Barcelona, Spain (Online), 2020). 10.18653/v1/2020.coling-main.481.

[28] Liu P (2023). Pre-train, Prompt, and Predict: A Systematic Survey of Prompting Methods in Natural Language Processing. ACM Comput. Surv..

[29] Cui, Y., Yang, Z. & Yao, X. Efficient and *Effective Text Encoding for Chinese LLaMA and Alpaca*. arXiv.org https://arxiv.org/abs/2304.08177v2 (2023).

[30] Bai, J. *et al.* Qwen technical report. *arXiv.org* https://arxiv.org/abs/2309.16609v1 (2023).

[31] Yang, A. *et al.**Baichuan 2: Open Large-scale Language Models*. Preprint at 10.48550/arXiv.2309.10305 (2023).

[32] Huang, Y. *et al.* C-Eval: *A Multi-level Multi-discipline Chinese Evaluation Suite for Foundation Models*. Preprint at 10.48550/arXiv.2305.08322 (2023).

[33] Hendrycks, D. *et al.**Measuring Massive Multitask Language Understanding*. Preprint at http://arxiv.org/abs/2009.03300 (2021).

[34] Loshchilov, I. & Hutter, F. *Fixing Weight Decay Regularization in Adam*. (2018).

[35] Gu Y (2021). Domain-specific language model pretraining for biomedical natural language processing. ACM Trans. Comput. Healthcare.

[36] Bellan P, Dragoni M, Ghidini C, Almeida JPA (2022). Extracting business process entities and relations from text using pre-trained language models and in-context learning. Enterprise Design, Operations, and Computing.

[37] Liu W, Lu W, Huang S, Hong Y, Zhou X (2022). An entity-centric medical consultation dataset for entity-aware medical dialogue generation. Natural Language Processing and Chinese Computing.

[38] Tianchi. *Entity Recognition of Traditional Chinese Medicine’s Manual*. https://tianchi.aliyun.com/dataset/dataDetail?dataId=86819 (2020).

[39] Xu, C., Guo, D., Duan, N. & McAuley, J. Baize: An open-source chat model with parameter-efficient tuning on self-chat data. Preprint at 10.48550/arXiv.2304.01196 (2023).

[40] Zeng, A. *et al.* GLM-130B: An open bilingual pre-trained model. Preprint at 10.48550/arXiv.2210.02414 (2022).

[41] Du, Z. *et al.* GLM: General language model pretraining with autoregressive blank infilling. Preprint at 10.48550/arXiv.2103.10360 (2022).

[42] Bommasani, R., Liang, P. & Lee, T. Holistic evaluation of language models. *Annals of the New York Academy of Sciences* **1525**(1), 140–146 (2023). 10.1111/nyas.15007 37230490

[43] Ding N (2023). Parameter-efficient fine-tuning of large-scale pre-trained language models. Nat. Mach. Intell..

[44] Liu H (2022). Few-shot parameter-efficient fine-tuning is better and cheaper than in-context learning. Adv. Neural Inf. Process. Syst..

[45] Houlsby, N. *et al.* Parameter-efficient transfer learning for NLP. In *Proceedings of the 36th International Conference on Machine Learning* 2790–2799 (PMLR, 2019).

[46] Li, X. L. & Liang, P. Prefix-tuning: Optimizing continuous prompts for generation. Preprint at 10.48550/arXiv.2101.00190 (2021).

[47] Liu, X. *et al.* GPT Understands, Too. Preprint at 10.48550/arXiv.2103.10385 (2021).

[48] Liu, X. *et al.* P-Tuning v2: Prompt tuning can be comparable to fine-tuning universally across scales and tasks. Preprint at 10.48550/arXiv.2110.07602 (2022).

[49] Hu, E. J. *et al.* LoRA: Low-rank adaptation of large language models. Preprint at 10.48550/arXiv.2106.09685 (2021).

[50] Wei, J. *et al.* Finetuned language models are zero-shot learners. Preprint at 10.48550/arXiv.2109.01652 (2022).

[51] Wang, Y. *et al.* Self-Instruct: Aligning language models with self-generated instructions. Preprint at 10.48550/arXiv.2212.10560 (2023).

[52] Papineni, K., Roukos, S., Ward, T. & Zhu, W.-J. Bleu: a Method for automatic evaluation of machine translation. In *Proceedings of the 40th Annual Meeting of the Association for Computational Linguistics* 311–318 (Association for Computational Linguistics, Philadelphia, Pennsylvania, USA, 2002). 10.3115/1073083.1073135.

[53] Yuan, W., Neubig, G. & Liu, P. BARTScore: Evaluating generated text as text generation. Preprint at http://arxiv.org/abs/2106.11520 (2021).

[54] Lewis, M. *et al.* BART: Denoising sequence-to-sequence pre-training for natural language generation, translation, and comprehension. In *Proceedings of the 58th Annual Meeting of the Association for Computational Linguistics* (eds. Jurafsky, D., Chai, J., Schluter, N. & Tetreault, J.) 7871–7880 (Association for Computational Linguistics, Online, 2020). 10.18653/v1/2020.acl-main.703.

